# Nasopharyngeal tuberculosis: A case report

**DOI:** 10.1515/biol-2022-0077

**Published:** 2022-08-17

**Authors:** Yang Yang, Yuan Fang, GuoNing Yang

**Affiliations:** Department of Otorhinolaryngology Head and Neck Surgery, Baoshan People’s Hospital, Baoshan 678000, Yunnan Province, China; Department of Organ Transplantation, The First Affiliated Hospital of Kunming Medical University, 295 Xichang Road, Kunming 650032, Yunnan Province, China

**Keywords:** nasopharynx, tuberculosis, sore throat, histopathology

## Abstract

Nasopharyngeal tuberculosis is a rare disease. Even in areas where tuberculosis is endemic, its incidence is also extremely low. Here, we will report a rare case of nasopharyngeal tuberculosis. A 29-year-old male patient presented with a 2-month history of sore throat, nasal congestion, dysphagia, and low-grade fever. Thickened mucosa in the posterior wall of the nasopharynx was shown from nasopharyngoscopy, covered with a thick, yellowish, purulent secretion that was not easily removed. Computed tomography of the nasopharynx showed thickening of the mucosa in the right and left bilateral walls and the posterior wall with indistinct margins. Histopathological examination diagnosed nasopharyngeal tuberculosis granuloma. Nasopharyngeal tuberculosis is rare and has atypical symptoms. When a new organism appears in the nasopharynx, it should be differentiated from tuberculosis, autoimmune diseases, and tumors, and a tissue biopsy of the new organism should be performed to make a final diagnosis based on histopathology.

## 1 Background

Tuberculosis is highly prevalent worldwide, mainly caused by *Mycobacterium tuberculosis* infection, and, according to the report of world health organization, there were 10 million cases in 2018 while 1.5 million people died from the disease [1]. Tuberculosis can involve all parts of the body. However, nasopharyngeal tuberculosis is extremely rare, and its incidence is far less than 1% of tuberculosis. According to statistics, tuberculosis manifesting in the head and neck accounts for 10–35% of extrapulmonary tuberculosis [2]. It is easy to misdiagnose that in terms of clinical manifestations, symptoms of nasopharyngeal tuberculosis are atypical, such as tinnitus, nasal obstruction, sore throat, dyspnea, dysphagia, and hoarseness, to name a few. Furthermore, nasopharyngeal tuberculosis is often associated with enlarged cervical lymph nodes and is highly susceptible to misdiagnosis as nasopharyngeal malignancy. Therefore, accurate diagnosis of nasopharyngeal tuberculosis seriously relies on pathological histology.

To date, there have been few reports of nasopharyngeal tuberculosis, and the disease remains a relatively uncommon condition. Here, we report a case of nasopharyngeal tuberculosis that presented with a sore throat, nasal congestion, dysphagia, and low-grade fever for 2 months.

## 2 Case presentation

**Figure 1:**
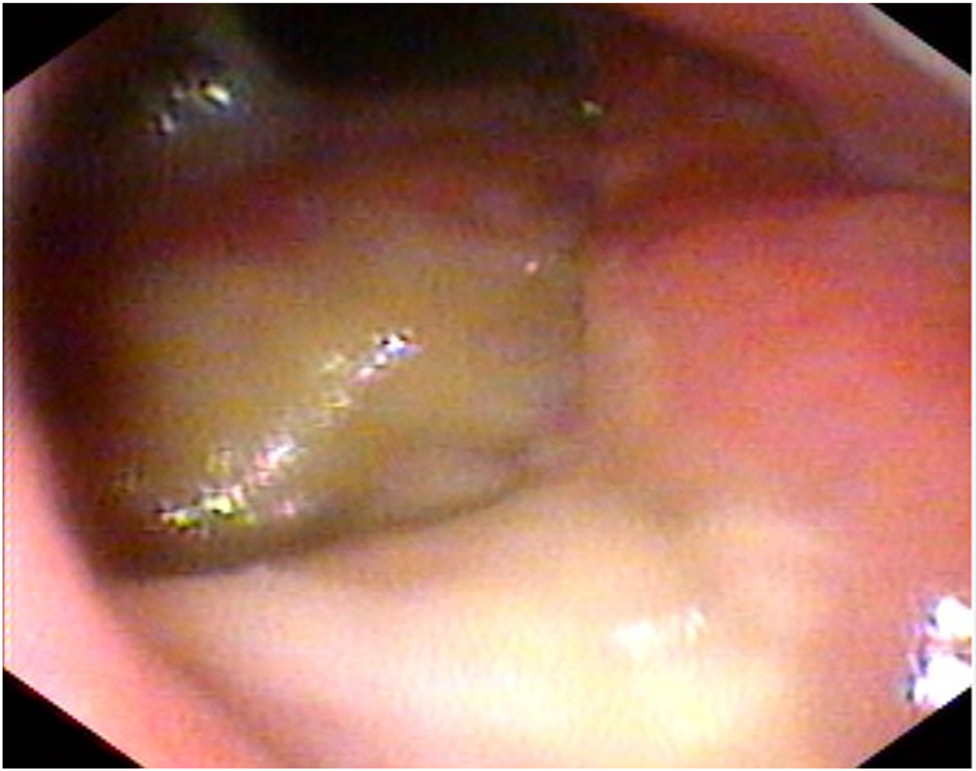
Nasopharyngoscopy showing thickened mucosa in the posterior wall of the nasopharynx covered with a thick, yellowish, purulent secretion that was not easily removed.

**Figure 2:**
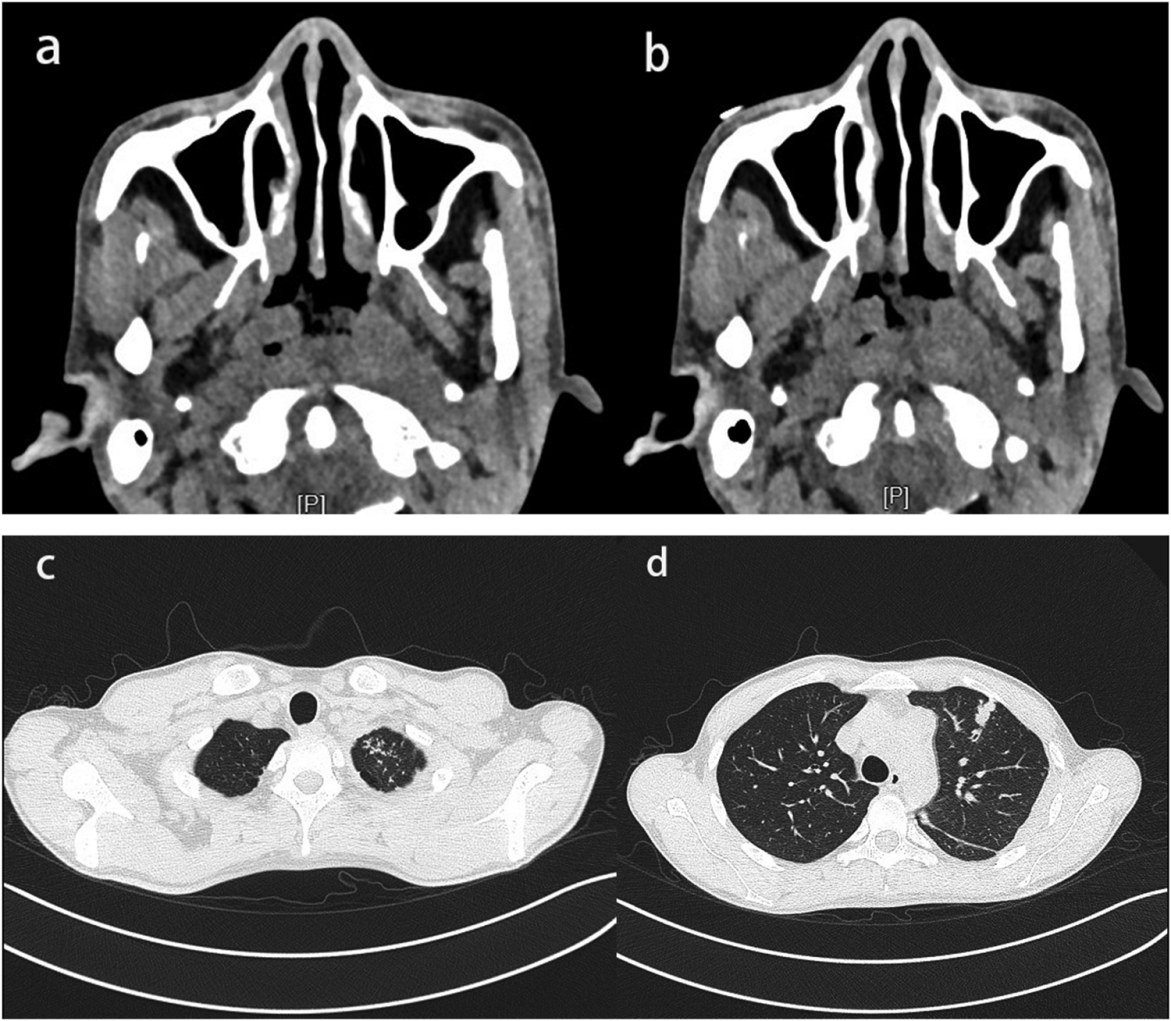
(a and b) Computed tomography of the nasopharynx showing thickening of the mucosa on the right and left side walls and posterior wall with unclear margins. (c and d) Axial computed tomography images of the chest demonstrating centrilobular nodules with tree-in-bud opacification involving apical and anterior segments of the left upper lobe. And showed left-sided pleural thickening.

**Figure 3:**
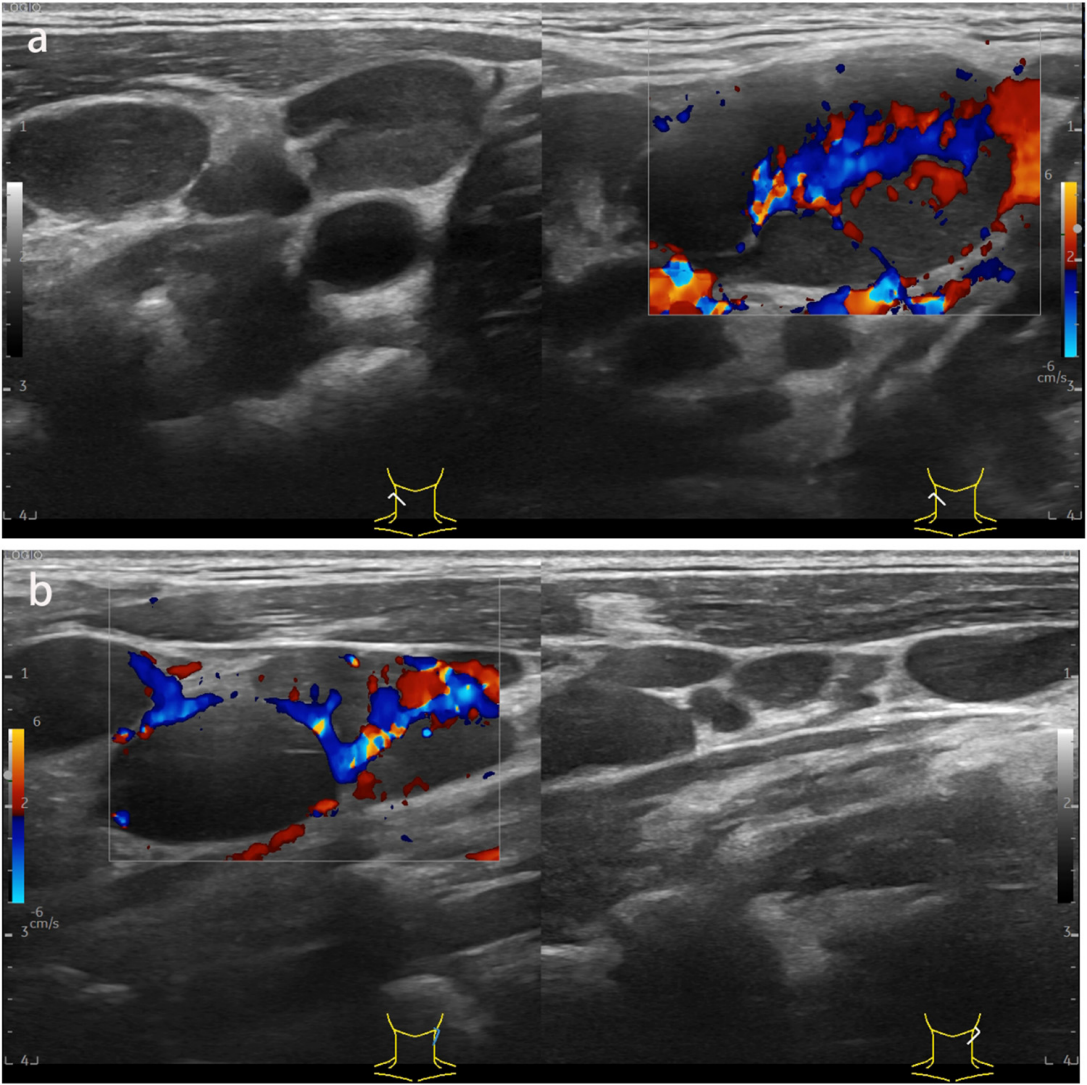
(a and b) Grey scale ultrasound images of the neck demonstrating multiple enlarged hypoechoic lymph nodes bilaterally with areas of partial liquefaction and necrosis.

**Figure 4:**
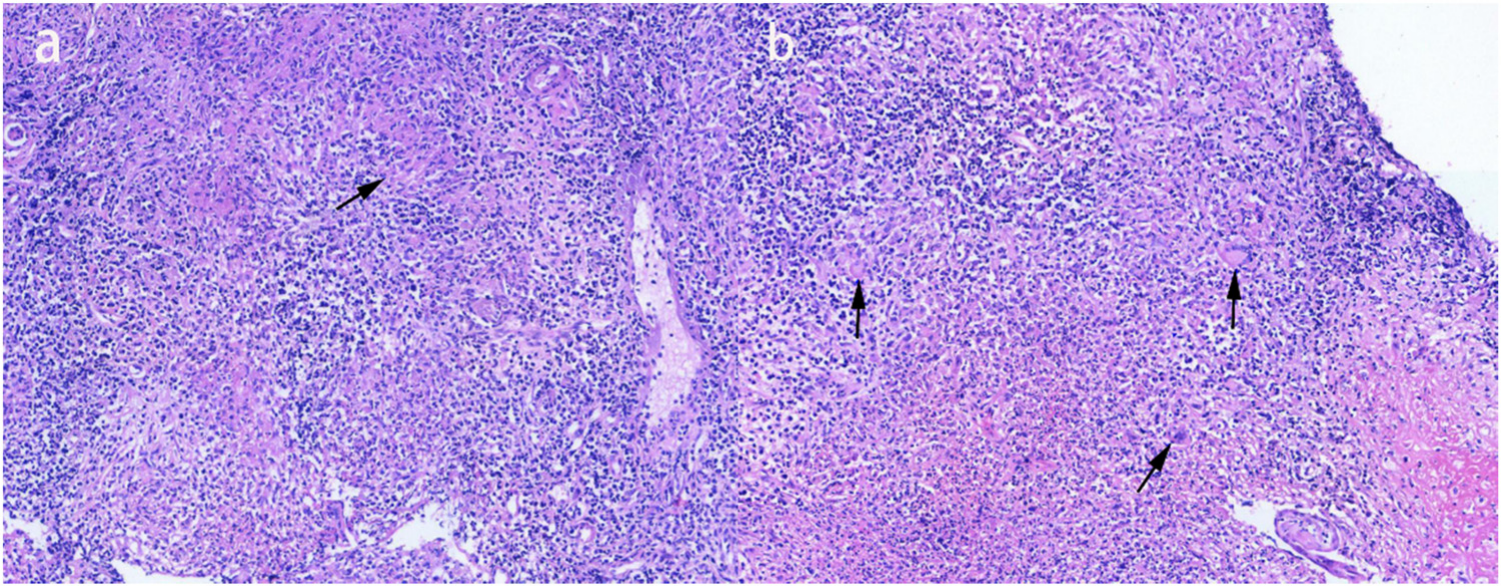
(a) Histopathological examination using hematoxylin–eosin staining at a microscopic magnification of 100× revealing epithelioid reaction. (b) Histopathological examination using hematoxylin–eosin staining at a microscopic magnification of 40× revealing multinucleated giant cells.


A 29-year-old male patient presented with a sore throat, nasal congestion, dysphagia, and low-grade fever for 2 months, with a temperature of up to 37.6°C. He denied a history of organ diseases such as diabetes, kidney disease, liver disease, tuberculosis infection, long-term use of hormones, immunosuppressants, and a family history of tuberculosis infection. Physical examination showed poor ventilation of the nasal cavity bilaterally, with hypertrophy and swelling of the nasal mucosa, purulent secretions, and multiple enlarged lymph nodes with clear borders, the larger one being about 2 cm × 3 cm, were palpated in the neck bilaterally. We took the secretion for culture and identification, which showed no pathogenic bacteria growth in bacterial culture and white pseudomycetes in fungal culture. In addition, nasopharyngoscopy showed thickened mucosa in the posterior wall of the nasopharynx, covered with a thick yellowish purulent secretion that was not easily removed (Figure 1). Computed tomography of the nasopharynx showed mucosal thickening on the left and right sides of the wall and posterior wall with indistinct margins and multiple enlarged lymph nodes in the retropharyngeal space, left the palatine tonsillar area, and both sides of the neck (Figure 2a and b). Computed chest tomography displayed pulmonary tuberculosis in the upper lobe of the left lung and left-sided tuberculous pleurisy (Figure 2c and d). Ultrasonography of cervical lymph nodes demonstrates multiple enlarged solid hypoechoic lymph nodes with well-defined borders, the largest on the right side measuring approximately 3.3 cm × 1.9 cm and the larger one on the left side measuring approximately 2.7 cm × 1.4 cm, with a few lymph nodes showing mildly increased vascularity and partial liquefaction (Figure 3a and b). Antacid bacilli can be found by taking sputum from patients for antacid bacillus smear examination. Moreover, we also performed a T-cell test for tuberculosis infection, whose result was positive. The above-mentioned tests suggested the possibility of nasopharyngeal tuberculosis in this patient, but a definitive diagnosis still required histopathological examination. Therefore, a nasopharynx extraction biopsy was performed using a 0° rigid nasal endoscope under local anesthesia. Postoperatively, the specimen was stained with hematoxylin–eosin, and epithelioid reaction (Figure 4a) was found under microscopic magnification of 100×, and multinucleated giant cells (Figure 4b) were observed under magnification of 40×, suggesting nasopharyngeal tuberculosis granuloma. Therefore, the patient was finally diagnosed with nasopharyngeal tuberculosis granuloma and tested for *Mycobacterium tuberculosis* rifampin resistance by sputum, which showed that the rifampin resistance mutation gene rpoB was sensitive. Furthermore, the *Mycobacterium tuberculosis* gene probe A–E (representing 81 bases in the RRDR region of the tuberculosis rpoB gene and 5 different DNA fragments) was positive, and the patient was given HRZE (isoniazid 300 mg, rifampin 450 mg, pyrazinamide 1,500 mg, ethambutol 750 mg) anti-tuberculosis treatment. After 1 month of antituberculosis treatment, the patient’s sore throat, nasal congestion, and dysphagia disappeared.

**Informed consent:** Informed consent has been obtained from all individuals included in this study.
**Ethical approval:** The research related to human use has been complied with all the relevant national regulations, institutional policies and in accordance with the tenets of the Helsinki Declaration, and has been approved by the authors’ institutional review board or equivalent committee.

## 3 Discussion

Nasopharyngeal tuberculosis is caused by infection with *Mycobacterium tuberculosis*. Studies have shown two main mechanisms leading to nasopharyngeal tuberculosis: (1) direct infection with *Mycobacterium tuberculosis*. Due to the unique structure of the nasopharynx and the lack of filtration and cleaning, it is effortless for *Mycobacterium tuberculosis* to stay and accumulate here; (2) caused by the lymphatic system or blood system. There is a large amount of lymphatic tissue under the mucous membrane of the nasopharynx and a rich blood circulation system. *Mycobacterium tuberculosis* can enter the lymphatic and circulatory systems through an immune response, leading to the development of nasopharyngeal tuberculosis. Worldwide, tuberculosis has a high morbidity and mortality rate, which can accumulate throughout the body organs [3]. However, even where tuberculosis is endemic, nasopharyngeal tuberculosis is still rare [4]. The clinical presentation of nasopharyngeal tuberculosis is atypical and can be easily overlooked in clinical work. For example, Nakao et al. [5] reported nasopharyngeal tuberculosis presenting with posterior nasal drip and cough, and Özcan et al. [6] reported a 17-year-old female patient with nasopharyngeal tuberculosis admitted with neck swelling and hearing loss. Nasopharyngoscopy presentations have also been varied, such as no abnormalities in appearance, ulcerated erosions, masses, and covered white patches [2]. These manifestations of nasopharyngeal tuberculosis can be seen in nasopharyngeal cancer. In the analysis of misdiagnosis of otorhinolaryngological diseases, nasopharyngeal tuberculosis ranks first, which is extremely easy to be misdiagnosed as a nasopharyngeal malignant tumor. In clinical work, it can be distinguished by the following points: (1) nasopharyngeal tuberculosis is more often seen in the parietal wall of nasopharynx, while the preferred site of nasopharyngeal cancer is in the saphenous fossa; (2) nasopharyngeal tuberculosis seldom invades the deep part, not destroying the bone of skull base and invading the brain nerve, while nasopharyngeal cancer can progress to the deep side, undermining the bone of skull base and violating the tip of rock bone to cause damage to the brain nerve; (3) the enlarged lymph nodes of nasopharyngeal tuberculosis often appear in bunches or fusion and may have low-density necrosis, while the lymph nodes in the neck of nasopharyngeal cancer are often solid and necrosis is less common; (4) the lymph nodes of patients with nasopharyngeal tuberculosis become soft and shrink after anti-tuberculosis treatment, while the lymph nodes in the neck of patients with nasopharyngeal cancer gradually increase in size. Because the clinical manifestations and nasal endoscopy of nasopharyngeal tuberculosis are similar to those of nasopharyngeal carcinoma, nasopharyngeal carcinoma is an important differential diagnosis. In addition, another important differential diagnosis of nasopharyngeal tuberculosis is sarcoidosis. Sarcoidosis is a disease that can involve all organs of the body and is characterized by non-caseous necrotizing epithelioid cell granuloma, which may have clinical manifestations such as fever, anorexia, weight loss, dry cough, and dyspnea. Imaging of the disease may show symmetrical enlargement of bilateral hilar and mediastinal lymph nodes with or without intrapulmonary lattices, nodular or lamellar shadows. Because the clinical presentation, imaging, and histopathology of sarcoidosis are very similar to those of tuberculosis, care should be taken to differentiate it from this disease in the diagnosis of tuberculosis. Although the distinction between the two relies mainly on the histopathological features of caseous necrosis, because necrotic tissue sometimes cannot be stained, researchers have suggested that sarcoidosis can be rapidly distinguished from tuberculosis by using next-generation sequencing technology [7]. However, the symptoms of nasopharyngeal lymphangiectasia are also non-specific, which need to be distinguished. Since they cannot be identified by the naked eye, a definitive diagnosis can only be made by relying on tissue biopsy [8]. In addition, nasopharyngeal tuberculosis also needs to be differentiated from fungal infection (aspergillus, mucormycosis), autoimmune disease (Wegener granulomatosis, polyarteritis nodosa, Churg–Strauss syndrome), and granulomatous inflammation (sarcoidosis, syphilis, leprosy, midline lethal granuloma) [2].

Treatment of nasopharyngeal tuberculosis is the same as that of pulmonary tuberculosis [2]. Anti-tuberculosis treatment for nasopharyngeal tuberculosis is effective. Patients’ clinical symptoms may disappear within 1 month, and the thickened mucous membrane in the nasopharynx may subside within 3 months, but without anti-tuberculosis treatment, *Mycobacterium tuberculosis* can spread to other tissues and organs, causing necrosis, which can even be life-threatening. For example, the middle ear can be involved through the eustachian tube, blood circulation, and lymphatic system, leading to tuberculous otomastoiditis. In addition, it may also lead to facial nerve palsy. Sebastian et al. [9] found that the incidence of facial nerve palsy was as high as 40% in a retrospective study of ten cases diagnosed with tuberculous otitis media. It has been shown that after 6 months of anti-tuberculosis treatment, there is no recurrence or growth in the following 7 years of follow-up [10]. In the present case, the patient required anti-tuberculosis treatment with HRZE (isoniazid 300 mg, rifampicin 450 mg, pyrazinamide 1,500 mg, ethambutol 750 mg) regimen for 12 months.

## 4 Conclusion

Nasopharyngeal tuberculosis is rare, though its incidence rate is also meager in areas where tuberculosis is endemic. In our case of nasopharyngeal tuberculosis reported here, although the admission examination suggested the possibility of nasopharyngeal tuberculosis, histopathological examination was still required to confirm if the patient was finally diagnosed with nasopharyngeal tuberculosis granuloma. Since the clinical manifestations of nasopharyngeal tuberculosis are atypical, and the nasopharyngoscopy is non-specific, it can easily lead to misdiagnosis. Moreover, it is difficult to distinguish nasopharyngeal tuberculosis from nasopharyngeal malignant tumors by the naked eye alone. Therefore, performing a biopsy of the nasopharyngeal tissue is particularly important. Nasopharyngeal tuberculosis can be confirmed by finding caseous necrosis in the pathological tissue.
